# Integrated analysis of DNA methylation and gene expression reveals specific signaling pathways associated with platinum resistance in ovarian cancer

**DOI:** 10.1186/1755-8794-2-34

**Published:** 2009-06-08

**Authors:** Meng Li, Curt Balch, John S Montgomery, Mikyoung Jeong, Jae Hoon Chung, Pearlly Yan, Tim HM Huang, Sun Kim, Kenneth P Nephew

**Affiliations:** 1 Medical Sciences, Indiana University School of Medicine, Bloomington, IN 47405, USA; 2 Indiana University Melvin and Bren Simon Cancer Center, Indianapolis, IN 46202, USA; 3 Department of Cellular and Integrative Physiology, Indiana University School of Medicine, Indianapolis, IN 46202, USA; 4 Department of Biological Sciences, Korea Advanced Institute of Science and Technology, 373-1 Guseong-dong, Yuseong-gu, Taejon 305-701, South Korea; 5 Division of Human Cancer Genetics, Comprehensive Cancer Center, The Ohio State University, Columbus, OH 43210, USA; 6 School of Informatics, Indiana University, Bloomington, IN 47405, USA; 7 Center for Genomics and Bioinformatics, Indiana University, Bloomington, IN 47404, USA; 8 Department of Obstetrics and Gynecology, Indiana University School of Medicine, Indianapolis 46202, USA

## Abstract

**Background:**

Cisplatin and carboplatin are the primary first-line therapies for the treatment of ovarian cancer. However, resistance to these platinum-based drugs occurs in the large majority of initially responsive tumors, resulting in fully chemoresistant, fatal disease. Although the precise mechanism(s) underlying the development of platinum resistance in late-stage ovarian cancer patients currently remains unknown, CpG-island (CGI) methylation, a phenomenon strongly associated with aberrant gene silencing and ovarian tumorigenesis, may contribute to this devastating condition.

**Methods:**

To model the onset of drug resistance, and investigate DNA methylation and gene expression alterations associated with platinum resistance, we treated clonally derived, drug-sensitive A2780 epithelial ovarian cancer cells with increasing concentrations of cisplatin. After several cycles of drug selection, the isogenic drug-sensitive and -resistant pairs were subjected to global CGI methylation and mRNA expression microarray analyses. To identify chemoresistance-associated, biological pathways likely impacted by DNA methylation, promoter CGI methylation and mRNA expression profiles were integrated and subjected to pathway enrichment analysis.

**Results:**

Promoter CGI methylation revealed a positive association (Spearman correlation of 0.99) between the total number of hypermethylated CGIs and GI_50 _values (*i.e*., increased drug resistance) following successive cisplatin treatment cycles. In accord with that result, chemoresistance was reversible by DNA methylation inhibitors. Pathway enrichment analysis revealed hypermethylation-mediated repression of cell adhesion and tight junction pathways and hypomethylation-mediated activation of the cell growth-promoting pathways PI3K/Akt, TGF-beta, and cell cycle progression, which may contribute to the onset of chemoresistance in ovarian cancer cells.

**Conclusion:**

Selective epigenetic disruption of distinct biological pathways was observed during development of platinum resistance in ovarian cancer. Integrated analysis of DNA methylation and gene expression may allow for the identification of new therapeutic targets and/or biomarkers prognostic of disease response. Finally, our results suggest that epigenetic therapies may facilitate the prevention or reversal of transcriptional repression responsible for chemoresistance and the restoration of sensitivity to platinum-based chemotherapeutics.

## Background

Ovarian cancer is the most deadly gynecological malignancy, with an overall U.S. five-year survival rate of only 46% [1]. While highly curable if diagnosed in the early (ovary-confined) stages, over 75% of initial diagnoses are Stage III or IV malignancies, for which the survival index is only 30.6% [1]. While most patients initially respond to surgical debulking and treatment with taxanes combined with platinum-based chemotherapies [2,3], over 80% of those responders eventually relapse with fully chemoresistant disease [4]. While a number of signal transduction cascades have been hypothesized to contribute to this devastating clinical phenomenon, the mechanism(s) underlying the onset of chemoresistance remains poorly understood, reviewed in [5].

Similar to most chemotherapies, the antitumor activity of cisplatin is dependent upon DNA damage of rapidly dividing cells, and is mediated primarily by the formation of intra- and interstrand cisplatin-DNA adducts [6]. The resulting accumulation of these DNA lesions is believed to lead to steric obstruction of DNA-binding proteins necessary for vital intracellular functions, including transcription and DNA replication, with recognition of the resulting lesions by high mobility group and mismatch repair proteins eventually leading to p53-initiated apoptosis [7]. Thus, drug inactivation, decreased accumulation of DNA-cisplatin adducts, defective DNA damage recognition, enhanced nucleotide-excision repair, and impaired apoptotic responses are hypothesized as broad-based mechanisms responsible for the drug-resistant phenotype [5,8,9]. While dysregulation of genes and pathways is often due to various rearrangements (*e.g*., deletions, mutations, or translocations) to the DNA molecule itself, epigenetic changes (*e.g*., DNA methylation and histone modifications) are likely even more prominent in the onset of chemoresistance [10-14]. Specifically, transcriptional silencing of distinct DNA repair and apoptosis-associated genes by hypermethylation of promoter "CpG islands" (CGIs), CG-rich DNA regions typically unmethylated in normal cells [15], has now been associated with platinum drug resistance in numerous cancers, including ovarian [9,16-21]. Moreover, the degree of aberrant methylation (*i.e*., the total number of methylated genes) has also been directly correlated with ovarian tumor progression and recurrence, and specific methylated loci have been statistically associated with poor progression-free survival in ovarian cancer [22-24]. However, no previous global studies of the accumulation of DNA methylation aberrations, during the gradual acquisition of chemoresistance, or their likely impact on specific biological signaling pathways, have been reported in cancer.

To identify epigenetically regulated genes directly associated with ovarian cancer cisplatin resistance, and their associated biological pathways, we established a cell culture model to emulate the time-dependent development of drug resistance in patients suffering from this condition. In this model, a single clone of the platinum-sensitive ovarian cancer cell line A2780 was exposed to incrementally increasing doses of cisplatin, generating A2780 sublines having varying degrees of chemoresistance. By categorizing distinct aberrations in DNA methylation and gene expression associated with specific time-points during the development of resistance, we demonstrated statistically significant correlations between promoter CpG island methylation and gene expression changes, and also between methylation and drug resistance, with consequent alterations in specific drug-response signaling pathways. In accord with a causal role for aberrant DNA methylation in cisplatin resistance, treatment of the drug-resistant sublines with DNA methylation inhibitors resulted in significant promoter demethylation and the re-establishment of chemosensitivity. While other studies have profiled gene expression [13,25,26], proteomic [27,28], and chromosomal aberrations [29,30] related to ovarian cancer cisplatin resistance, we believe this is the first report integrating chemoresistance-associated alterations in DNA methylation and gene expression to determine likely epigenetically regulated biological pathways related to drug sensitivity. Based on these results, we suggest that aberrant DNA methylation may contribute to the disruption of key biological pathways during ovarian tumor progression to a drug-resistant phenotype. We believe our findings justify further cellular and molecular biologic studies for the development of more effective approaches for the clinical use of platinum-based chemotherapeutics.

## Methods

### Cell lines, drug treatments, and cell proliferation assays

All cells were maintained in RPMI 1640 media with 2 mM L-glutamine, 50 U/ml penicillin, 50 mg/ml streptomycin, and 10% fetal bovine serum, at 30°C and 5% CO_2_. 5-aza-2'-deoxycytidine (5-aza-dC) was purchased from Sigma (St. Louis, MO) and zebularine [1-(β-D-ribofuranosyl)-1,2-dihydropyrimidin-2-one] was a kind gift from Dr. Victor Marquez (Developmental Therapeutics Program, National Cancer Institute, Frederick, MD). A2780 ovarian cancer cells were obtained from ATCC (Manassas, VA), restriction enzymes from New England Biolabs (Beverly, MA), and cell culture reagents from Invitrogen (Carlsbad, CA). Using serial dilution cell seeding, a single clone of the cisplatin-sensitive, epithelial ovarian cancer cell line A2780 was cultured for multiple cycles ("treatment rounds") with incrementally increasing doses of cis-diamminedichloroplatinum(II) dichloride (CDDP, cisplatin) (Sigma). 5-aza-dC or zebularine treatment was performed after cell seeding for 48 hours prior to cisplatin treatment [31]. MTT assays were used to determine both GI_50 _values and growth curves of the cells, as we have described previously [31]. Briefly, 96-well dishes were plated with 2,000 cells per well one day before cisplatin treatment. The next day, cells were treated with various dosages of cisplatin (0.5 μM to 100 μM) for three hours and allowed to recover for three days. Following drug treatments, total viable cell numbers were determined by 4-hr treatments with 3-(4.5-dimethylthiazol-2-yl)-2.5-diphenyl tetrazolium bromide (MTT) assay, with cell viability (as determined by MTT metabolism to formazan) determined by measuring absorbance at 600 nm using a Bio-Tek (Winooski, VT) microplate spectrophotometer. Dose-response curves were then generated and 50% growth inhibitory (GI_50_) dose values determined by Microsoft Excel or Prism 4.0 (GraphPad Software, San Diego, CA), using sigmoidal dose (variable slope) curve fitting, as we have described previously [31,32].

### Differential methylation hybridization and data processing

Genomic DNA was isolated from parental (drug-sensitive) A2780, Round1, Round3, and Round5 cells using DNeasy purification kits (Qiagen, Valencia, CA). Differential methylation hybridization (DMH) was then performed as previously described [22,33,34]. Briefly, isolated DNA was digested with the methylation-insensitive restriction enzyme BfaI (C^TAG), followed by ligation of linkers. Linker-ligated DNA was then digested by the methylation sensitive enzymes HinP1I (G^CGC) and HpaII (C^CGG), and digestion products were then amplified by linker PCR. The PCR products were further amplified using aminoallyl-dUTP incorporation to facilitate labeling with the fluorophores Cy3 (parental A2780) or Cy5 (A2780 following various "rounds" of cisplatin treatment). The labeled DNA samples were then combined and hybridized to a customized 60-mer oligo microarray containing 40,000 CpG-rich fragments from 12,000 known gene promoters (Agilent, Santa Clara, CA) (see Additional file 1 for full annotations). Following hybridization and washing, microarray images were scanned and generated using an Axon GenePix 4200A scanner (Molecular Devices, Sunnyvale, CA). The raw microarray data was first corrected for background noise by background subtraction and then for system and technical noise by LOESS normalization. The relative methylation levels (*i.e*., "folds-change"), for each hybridized probe, in cisplatin-resistant sublines, were approximated by the ratios of the Cy5 (drug-treated cell lines) to Cy3 (parental A2780 cell line) fluorescence, and were defined as positive or negative values according to their respective increases or decreases compared to the parental cells. To avoid potential technical variations between probes, the methylation levels of multiple probes for a single gene (average of four probes per gene) were not collapsed or averaged, and genes having multiple discordant probe intensities were eliminated from the analysis. The DMH data have been deposited in NCBI Gene Expression Omnibus (GEO, ) and are accessible through GEO SuperSeries GSE15709.

### Gene expression microarray analysis and data processing

For gene expression assessments, total RNA was isolated from parental A2780 cells and Round5 cells using Qiagen RNeasy purification kits (Valencia, CA) and further purified with RNase-free DNase following our previously described method [32]. All microarray hybridizations were performed at the Indiana University Center for Medical Genomics (IUCMG). Five replicates were performed for each cell line using Human U133 plus 2.0 GeneChips (Affymetrix, Santa Clara, CA). Using Bioconductor [35], present (P), absent (A) or marginal (M) calls were determined using an MAS5 algorithm. Fraction presence, defined as the average present/absent (P/A) detection call (scores were given as P = 1, M = 0.5 and A = 0) for the experimental or control groups, was calculated for each microarray probe, and probes with at least one group having a fraction presence of 0.5 were selected for future use. Welch's t-test was performed for each probe using their log-transformed signals, with p-values less than 0.01 considered significant. To further support the statistical significance of probes having p < 0.01, the false discovery rate (FDR) was also calculated [36,37], with probe significance defined as an FDR of less than 5%. A moderately stringent fold-change cutoff of ≥ 1.5 (or ≤ -1.5 for downregulation), which allows for an acceptable balance between false discovery and false negative rates [38], was applied (in addition to the p-value cutoffs) to determine genes with significant expression alterations. The gene expression microarray data have been deposited in NCBI Gene Expression Omnibus (GEO, ) and are accessible through GEO SuperSeries GSE15709.

### Clustering, functional pathway prediction, gene ontology (GO) analysis, and statistics

Hierarchical clustering of the expression profiles was done by Cluster 3.0 [39]. Expression profiles were first pre-filtered by fraction present and then input into the program. The input data was further log transformed and normalized by array median centering and gene median centering. Average linkage clustering was performed by correlation (centered) similarity metric. Clustering results were viewed using TreeView version 1.60 [39]. The online program Pathway-Express (Onto-Tools, Wayne State University, Detroit, MI) [40,41] was used to explore the most biologically relevant pathways impacted by a list of input genes. Specific biological pathways were defined by the Kyoto Encyclopedia of Genes and Genomes (KEGG) database (Kanehisa Laboratories, Japan) [42]. Given a list of genes (for example, upregulated genes in Round5), Pathway-Express selects pathways based on impact analysis that considers not only conventional statistical analysis but also other biological factors, such as expression levels (*i.e*., fold-change) of input genes, type and position in a given pathway, and protein-protein interactions, among other variables. Thus, this approach is considered to be more powerful than analyses based on statistics only. Corrected p-values of less than 0.05 were used as an empirical cutoff for retrieving altered pathways [41]. GO enrichment of expression profiles was also analyzed by the online program Functional Annotation Analysis Tools, provided by the Database for Annotation, Visualization and Integrated Discovery (DAVID) bioinformatics database (NIAID, NIH, Bethesda, MD) [43-45]. All statistics were done in R and Microsoft Excel, unless otherwise stated.

## Results

### Establishment of a cell culture model of acquired ovarian cancer platinum resistance

**Figure 1:**
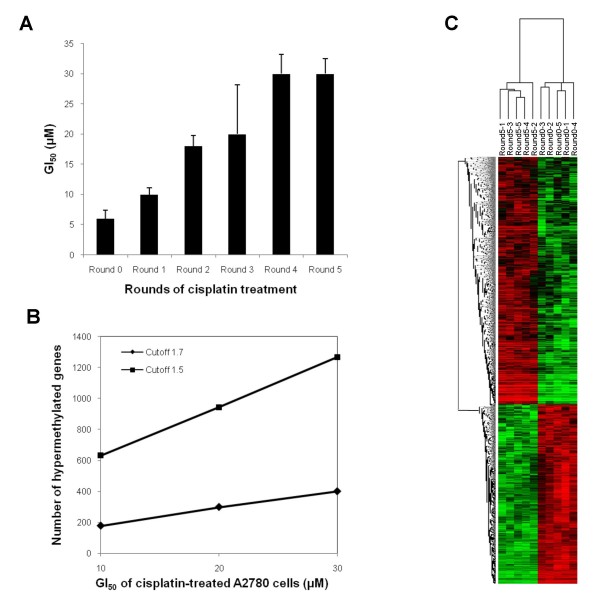
***De novo *DNA methylation and acquired cisplatin resistance in an ovarian cancer cell model**. A) Cisplatin-sensitive A2780 epithelial ovarian cancer cells were treated with 70% of GI_50 _dose of cisplatin. Surviving cells (Round1 A2780) were expanded and treated with the subsequent 70% GI_50 _dose (total of five rounds of drug treatment). The GI_50 _values for both parental and cisplatin-treated A2780 sublines were measured by MTT assay. B) DMH microarrays were performed on Round1, Round3 and Round5 A2780 cells, and the number of hypermethylated genes for each round was determined using various fold-change cutoffs (1.5-fold or 1.7-fold) and plotted as a function of cisplatin GI_50_. The correlations were determined by Spearman correlation. C) mRNA expressions in parental (cisplatin-sensitive) and Round5 (cisplatin-resistant) A2780 cells were measured by Human U133 plus 2.0 GeneChips (Affymetrix, Santa Clara, CA). Two-dimensional hierarchical clustering of the 3127 significantly up- or down-regulated probes done by Cluster [39] revealed distinctively different mRNA expression profiles of the two A2780 sublines (detailed plot provided in Additional file 2).

To establish a model for the development of ovarian tumor cisplatin resistance, we exposed clonally derived, platinum-sensitive A2780 ovarian cancer cells [46] to incrementally increasing doses of cisplatin, with drug sensitivity assessed by MTT cell proliferation assays. The cisplatin GI_50 _dose (*i.e*., dose necessary for 50% growth inhibition) for the starting clone of A2780 cells was 5 μM (Figure 1A), and these starting cells were designated as "Round0" cells, to denote their parental relationship to their subsequent drug-selected progeny. Parental A2780 cells were then treated with cisplatin at 70% of their GI_50 _dose (initially at 5 μM) for 3 hours and then allowed to recover for two weeks. The surviving cells were then expanded and designated as "Round1" cells, to denote their single cisplatin-selection cycle. The same procedure was repeated four additional times to generate "Round2" to "Round5" cisplatin-selected A2780 cells. Following each selection cycle, the cisplatin GI_50 _dose for the surviving cells was determined, and was found to increase continuously to 35 μM for Round5 cells (Figure 1A), a dose similar to the commonly used cisplatin-resistant cell line A2780CP [47].

### Positive correlation between DNA methylation and cisplatin-resistance development

To examine changes in DNA methylation during the development of drug resistance, we utilized a genome-wide methylation microarray approach, differential methylation hybridization (DMH) [33], to compare CpG island (CGI) methylation profiles of: 1) Round1 (one cisplatin treatment) vs. parental A2780; 2) Round3 (three cisplatin treatments) vs. parental A2780; and 3) Round5 (five cisplatin treatments) vs. parental A2780. The methylation status of any specific gene was estimated by its ratio of normalized Cy5 to Cy3 fluorescent signal (*i.e*., fold-change). To select differentially methylated genes, a moderately stringent fold-change cutoff of 1.5, allowing for an acceptable balance between false discovery and false negative rates [38], was used (*i.e*., hypermethylation was defined as fold-change ≥ 1.5 and hypomethylation as fold-change ≤ -1.5). Using that cutoff limit, the total numbers of hypermethylated genes for the Round1, Round3 and Round5 sublines were 595, 870 and 1176, respectively, relative to the parental ("Round0") A2780 cells. Spearman correlation testing further demonstrated a significant and positive linear correlation between the total number of hypermethylated genes and the GI_50 _values of the cisplatin-resistant sublines (Figure 1B), in accord with previous studies demonstrating increasing CGI methylation during ovarian tumor progression [23,24].

**Figure 2:**
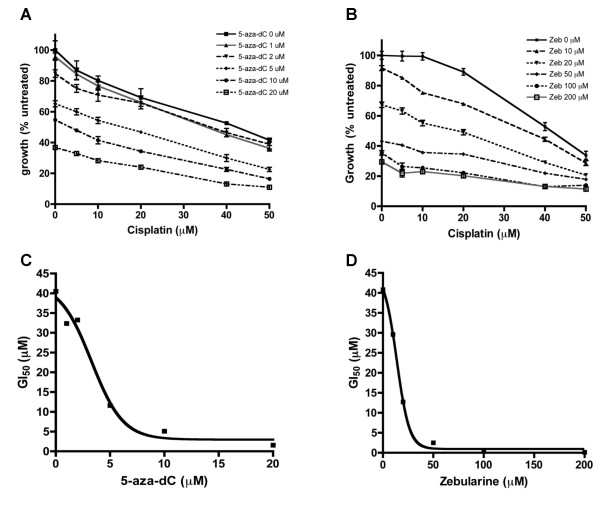
**Resensitization of cisplatin-resistant Round5 A2780 cells by 5-aza-dC and zebularine**. A) Growth curves for cisplatin in the presence of 5-aza-dC pretreatment. In the absence of cisplatin, higher 5-aza-dC dosage achieves higher cell death. The same dosage of cisplatin achieves higher cell death with higher dosages of 5-aza-dC, demonstrating increased cisplatin sensitivity by 5-aza-dC. B) The same resensitization experiment was performed for zebularine, using higher dosages (0–200 μM) [49,50]. C) The cisplatin GI_50 _values measured by MTT assay showed a dose-dependent decrease by 5-aza-dC. D) The cisplatin GI_50 _values measured by MTT assay showed a dose-dependent decrease by zebularine.

In agreement with our results showing progressively increasing total CGI methylation during the development of cisplatin resistance, we also observed upregulation of the DNA methyltransferases DNMT1 and DNMT3B in the drug-resistant Round5 subline (vs. the parental line), with expression fold-changes of 1.63 (p = 0.0011, FDR = 0.012) and 1.80 (p = 0.0004, FDR = 0.007), respectively (Table 1). To further examine the involvement of DNA methylation in drug resistance, the Round5 subline was treated with various doses of two routinely used DNA methyltransferase inhibitors, 5-aza-2'-deoxycytidine (5-aza-dC, decitabine) and zebularine [48]. 5-aza-dC (10 μM) treatment alone resulted in a 45% decrease in total cell number, while 10 μM zebularine alone resulted in 10% cell death (Figures 2A, 2B). The Round5 subline was then pretreated for 48 hr with 5-aza-dC or zebularine prior to administration of the GI_50 _dose of cisplatin. As shown in Figure 2A, 10 μM 5-aza-dC pretreatment, followed by cisplatin exposure, resulted in a marked decrease in cell survival. Furthermore, cisplatin sensitivity increased with each dosage of 5-aza-dC, as summarized in Figure 2C, demonstrating decreasing cisplatin GI_50 _as a function of 5-aza-dC dose. Similar resensitization results were observed after treatment of the Round5 subline with zebularine (Figures 2B, 2D), although a higher dose of zebularine was required, due to the lower drug potency of that agent [49,50]. Consequently, these results indicate a role for aberrant DNA methylation in the reduced sensitivity of ovarian cancer cells to cisplatin-mediated DNA damage, and suggest that DNA methyltransferase inhibitors represent one possible strategy for chemosensitization.

**Table 1:** mRNA expression level changes of DNA methyltransferases (DNMTs) in Round5 vs. parental A2780 cells

**Gene**	**Welch's t-test**	**FDR**	**Fold-change**	**Gene title**
DNMT1*	0.0011	0.0123	1.63	DNA (cytosine-5-)-methyltransferase 1
DNMT2	0.3673	0.5360	1.17	DNA (cytosine-5-)-methyltransferase 2
DNMT3A	0.1009	0.2275	1.20	DNA (cytosine-5-)-methyltransferase 3 alpha
DNMT3B *	0.0004	0.0069	1.80	DNA (cytosine-5-)-methyltransferase 3 beta

* Genes with significant alteration in their mRNA expression

### Gene expression profiles reveal cisplatin-induced alterations in drug-resistant ovarian cancer

To identify changes in gene expression associated with cisplatin resistance (in possible correlation with promoter DNA methylation), microarray studies were performed on the Round5 subline (chemoresistant) vs. the parental line (A2780; chemosensitive). Total RNA was isolated from Round5 and parental (Round0) cells, labeled and hybridized to Human U133 plus 2.0 Affymetrix microarrays. Probes with fraction presence (see methods) < 0.5 were removed. The remaining 23,723 probes were then log-transformed and median-centered between arrays and within genes. Unsupervised hierarchical clustering (average linkage clustering method) was then utilized to visualize common expression patterns between the samples [39], demonstrating clustering of all five replicates of the Round5 subline gene expression microarray results, while analogously, the five replicate microarray results of the parental line formed a separate cluster. Figure 1C and Additional file 2 showed the clustering results of all (3127) significantly up- or down- regulated probes (p-value < 0.01, FDR < 5%, fold-change ≥ 1.5 or ≤ -1.5), these probes corresponded to a total of 1036 genes upregulated and 1286 genes downregulated in Round5 (see Additional file 3). The functional clustering analysis, an algorithm capable of clustering similar annotations across several often-used annotation types (*e.g*., GO terms, pathways, Swiss-Prot knowledgebase keywords) or within different levels of annotation terms (to highlight functional changes), provided by DAVID (Database for Annotation, Visualization and Integrated Discovery, developed by NIAID/NIH) [43-45], was then used to investigate gene ontology (GO) [51] term enrichment. Functional clustering analysis of all 1036 genes upregulated in Round5, using a high classification stringency (Kappa similarity term overlap ≥ 3 and threshold of 0.85; classification multiple linkage threshold of 0.5), showed 31 functional groups to be highly enriched (geometric p-values < 0.01), with cell cycle progression, cellular metabolic processes and DNA damage response as the top three ranked GO biological process terms (see Additional file 4). Conversely, using the same high classification stringency, all 1286 downregulated genes segregated into 16 enriched functional groups, with cellular metabolic processing, biological regulation, macromolecule and protein metabolic processes and positive regulation of transcription as the highest ranking GO biological pathway terms (see Additional file 5).

### Cisplatin resistance is associated with promoter CpG island DNA methylation of key genes in vital intracellular pathways

To investigate the potential functional role of promoter methylation in the onset of cisplatin resistance, pathway enrichment analyses were performed using Pathway-Express [40,41], a program that processes a list of input genes to identify KEGG pathways (Kanehisa Laboratories, Japan) [42] likely altered by those genes based on fold-change, pathway position, and pathway topology. As total promoter CGI hypermethylation was significantly associated with gene silencing (Figure 1B), we first selected genes that were coordinately hypermethylated and downregulated in Round5 as the Pathway-Express input list. Of the 1176 genes hypermethylated in Round5 cells (as compared to the parental A2780 cells) on the Agilent custom promoter CGI microarray, 847 were also annotated on the Affymetrix Human U133 plus 2.0 GeneChips. Of those 847 genes, 55 were found to be significantly downregulated (denoted as "gene list 1"). A second gene list, possessing all Round5 1,286 downregulated genes (regardless of methylation status), was then utilized as a background for all possible downregulated pathways in the cisplatin-resistant cells ("gene list 2"). To evaluate the over-representation of methylation-regulated genes in specific pathways, and to examine relevant differences between numbers of genes in each pathway in gene lists 1 and 2, a one-tailed Fisher's exact test was performed to statistically determine whether a majority of the downregulated genes were also methylated. That analysis revealed that three downregulated pathways, cell adhesion molecules (CAMs), tight junction, PPAR signaling and leukocyte transendothelial migration pathways were dominantly regulated by hypermethylation of these 55 genes (Fisher's exact test p-value < 0.05) (Table 2).

**Table 2:** Biological pathways repressed by hypermethylation

Pathways	Downregulated genes (1286)		Hypermethylated and downregulated genes (55)			Fisher's Exact Test p-values^§^
			
	Input genes in pathway	Corrected p-value	Input genes in pathway	Genes in pathway	Corrected p-value	
Cell adhesion molecules	14	1.95E-164	4	ITFAV, CLDN11, NEO1, CDH2	0	0.002*
Tight junction	13	1.40E-03	3	CLDN11, PPP2R4, INADL	9.93E-07	0.016*
PPAR signaling pathway	6	1.17E-01	2	CPT1A, SLC27A6	5.20E-03	0.024*
Leukocyte transendothelial migratio'n	9	2.34E-01	1	CLDN11	2.51E-02	0.326

Pathways listed are all pathways regulated by hypermethylated and downregulated genes, determined by Pathway Express corrected p-value < 0.05.

^§ ^Pathway enrichment p-values were calculated using a one-tail Fisher's exact test. Asterisks show p-value < 0.05

Similar to our examination of genes that were coordinately hypermethylated and downregulated in Round5 cells, we performed the same analysis for Round 5 genes found jointly hypomethylated and upregulated. Those results revealed that six pathways, pancreatic cancer, prostate cancer, colorectal cancer, non-small cell lung cancers, glioma, melanoma, and chronic myeloid leukemia, were all upregulated by hypomethylation (Fisher's exact test p-value < 0.05) (Table 3). Hypomethylated and upregulated genes in these pathways included *PIK3R3*, *PDGFRA*, *E2F1*, and *TGFBR2*, all signal transduction regulators associated with the PI3K/Akt, cell cycle progression, and TGF-beta pathways, and shared by all the cancer-associated pathways mentioned above.

**Table 3:** Biological pathways activated by hypomethylation

Pathways	Upregulated genes (1036)		Hypomethylated and upregulated genes (55)			Fisher's Exact Test p-values^§^
			
	Input genes in pathway	Corrected p-value	Input genes in pathway	Genes in pathway	Corrected p-value	
Glioma	6	1.60E-02	3	PIK3R3, PDGFRA, E2F1	8.69E-05	0.003*
Melanoma	5	6.00E-02	3	PIK3R3, PDGFRA, E2F1	3.91E-04	0.001*
Pancreatic cancer	8	3.30E-02	3	PIK3R3, E2F1, TGFBR2	1.10E-03	0.006*
Prostate cancer	9	3.80E-02	3	PIK3R3, PDGFRA, E2F1	1.20E-03	0.009*
Colorectal cancer	6	1.84E-01	3	PIK3R3, E2F1, TGFBR2	1.50E-03	0.003*
Chronic myeloid leukemia	6	1.75E-01	3	PIK3R3, E2F1, TGFBR2	2.50E-03	0.003*
Non-small cell lung cancer	2	6.00E-01	2	PIK3R3, E2F1	2.46E-02	0.003*
Phosphatidylinositol signaling system	4	5.32E-07	1	PIK3R3	2.23E-12	0.196
Gap junction	5	4.12E-02	1	PDGFRA	1.06E-05	0.239
Focal adhesion	17	8.76E-03	2	PIK3R3, PDGFRA	9.13E-05	0.226
MAPK signaling pathway	8	5.90E-02	2	PDGFRA, TGFBR2	9.73E-05	0.063
TGF-beta signaling pathway	6	1.13E-01	1	TGFBR2	5.67E-04	0.280
Adherens junction	7	4.44E-02	1	TGFBR2	6.41E-03	0.318
Regulation of actin cytoskeleton	15	8.57E-02	3	PIK3R3, PDGFRA, MSN	6.73E-03	0.051
Calcium signaling pathway	5	2.65E-01	1	PDGFRA	1.70E-02	0.239
Leukocyte transendothelial migration	9	3.98E-02	2	PIK3R3, MSN	1.92E-02	0.078

Pathways listed are all pathways regulated by hypermethylated and downregulated genes, determined by Pathway Express corrected p-value < 0.05.

^§ ^Pathway enrichment p-values were calculated using a one-tail Fisher's exact test. Asterisks show p-value < 0.05

## Discussion

Platinum compounds have served as a standard therapy for post-surgical ovarian cancer patients for over two decades, as well as for other malignancies, including testicular, bladder, lung, endometrial, and head and neck cancers [52]. Acquired or *de novo *resistance to platinum-based chemotherapy is commonly observed in ovarian cancer, with numerous underlying mechanisms now proposed to explain this phenomenon, including drug inactivation, elevated resistance to apoptosis, decreased recognition of DNA damage, and increased DNA repair [8,9,53,54]. Accumulating evidence now shows that aberrant epigenetic alterations contribute to these chemoresistance-associated phenomena, perhaps even more so than genetic aberrations [10,16]. By comparing and combining genome-wide gene expression and methylation changes observed in cisplatin-sensitive and -resistant ovarian cancer sublines, we discovered both novel and reported pathways and gene ontology (GO) groups likely to mediate acquired cisplatin resistance. These included cell cycle progression (G2/M checkpoint), response to DNA damage, nucleotide binding, and various cellular metabolic processes, in agreement with previous reports [9] and our previous study [55], further supporting a role for promoter CpG island (CGI) methylation in disrupting gene expression during tumor progression.

One possible explanation for our observed chemoresistance-associated changes in DNA methylation patterns is aberrant activity or substrate specificity of DNA methyltransferase (DNMT) enzymes [56,57]. In the current study, we observed modest but highly significant upregulation of both *DNMT1 *and *DNMT3B *in the cisplatin-resistant Round5 (*i.e*., treated for five drug cycles) A2780 subline (Table 1), suggesting that the altered methylation profile in these cells may be associated with increased or altered DNMT activity. In support of this possibility, a number of other studies have demonstrated pharmacologic or genetic downregulation of *DNMT1 *and *DNMT3B *enhanced chemosensitivity to various platinum drugs, including cisplatin [31,58-61]. Our current results demonstrate that 5-aza-dC and another methylation inhibitor, zebularine, dose-dependently restored chemosensitivity to Round5 cells (Figure 2), and previously we reported upregulation of *DNMT1 *and *DNMT3B *in ovarian cancer cell lines, along with a potential positive correlation between *DNMT1 *overexpression and tumor aggressiveness [62]. Alterations in DNMT isoforms have also been reported in ovarian cancer, with a complex relationship between global DNA hypomethylation and regional hypermethylation [63,64], while one study of serous endometrioid cancer actually demonstrated DNMT downregulation [65].

In addition to aberrant DNMT enzyme activity, drug-induced *de novo *promoter methylation has been hypothesized [66]. This phenomenon is believed to be due to DNA structural distortions resulting from the formation of cisplatin-DNA adducts, allowing subsequent access of DNMTs and/or other auxiliary methylation machinery components to target DNA regions [67,68]. It has also been demonstrated that endogenous DNA damage can alter the site selectivity of DNMT1 or recruit DNMTs to sites of repair [69,70]. The fact that chemoresistance is reproducibly reversible by inhibition of DNA methyltransferases further suggests that patients whose cancers have methylation of multiple relevant genes might be selected as candidates for demethylating therapy, in addition to platinum-based ovarian cancer chemotherapy, an approach that our group (National Institutes of Health, NIH, Study NCT00477386) and others (NIH studies NCT00748527 and NCT00529022) are taking to the clinic [71].

Our promoter CGI methylation profiles from A2780 sublines representing early, intermediate and late-stage cisplatin resistance demonstrated that the total number of hypermethylated genes linearly increased (Spearman correlation 0.99) with increasing cisplatin resistance (Figure 1B). This positive correlation suggests that in concert with altered promoter DNA methylation, distinct methylation profiles progressively may emerge during the development of drug resistance. Our previous studies of methylation profiling of late stage ovarian cancer patient tumors correlated CGI methylation and disease recurrence [22,23], further supporting an association between progressive methylation patterns and advanced disease [24,72]. Such methylation patterns may disrupt specific intracellular signaling pathways, and while using genome-wide approaches to demonstrate direct regulation of gene expression by aberrant promoter CGI methylation has been a challenge in the epigenomics field, direct or indirect biological outcomes of epigenetic modifications commonly associate with specific cellular behavior changes.

To assess the role of promoter CGI methylation in cisplatin resistance, we examined biological pathways potentially dysregulated by hypermethylation (Table 2), showing likely DNA hypermethylation-downregulated pathways such as CAMs, tight junction formation, PPAR signaling, and leukocyte transendothelial migration pathways. CAMs and tight junctions, by affecting signal transduction pathways, are both directly involved in the regulation of cell proliferation, differentiation, and apoptosis [73,74], and loss of functional tight junctions has been associated with tumorigenesis [75]. Specifically, hypermethylation-associated downregulation of numerous claudins, integral membrane protein constituents of tight junctions, has been demonstrated to associate with tumorigenesis and tumor invasion in ovarian and other cancers, including those of the breast, bladder, and colon [76-80]. In addition to alterations in claudin and CAM functions, we observed hypermethylation and downregulation of gene products previously hypothesized as suppressors of ovarian tumor progression, including alpha-integrins (possible regulators of cell proliferation and adhesion), carnitine palmitoyltransferase I (CPT1A, a protein believed to play a role in histone deacetylase inhibition), and N-cadherin (CDH2), a member of the cell adhesion proteins often lost during tumor progression [81-84]. Consequently, using global approaches, our analyses identified several potential links between cell adhesion and the acquisition of chemoresistance, in the context of regulation by epigenetic modification.

By using a similar approach for hypermethylated and downregulated genes in the cisplatin-resistant cells, we identified several pathways likely regulated by promoter CGI hypomethylation, including those characteristic of other cancers, such has pancreatic, prostate, colorectal, non-small cell lung cancers, chronic myeloid leukemia, glioma, and melanoma (Table 3). Interestingly, all significantly enriched pathways were found to be cancer-related, and those broad-based cancer categories commonly included signal transduction pathways such as phosphatidylinositol kinase/Akt, transforming growth factor-beta, the E2F transcription factor family, and platelet-derived growth factor signaling (PDGFR). Specifically, the PI3K/Akt pathway has been shown to contribute to cisplatin resistance by promoting cell proliferation and increasing drug metabolism and resistance to apoptosis [85,86], while E2F transcription factors have been previously implicated in platinum-resistant ovarian cancer [87-89]. Moreover, while regulation of *TGFBR2 *by promoter CGI methylation has previously been reported in lymphoma [90], a role for promoter hypomethylation in dysregulation of that pathway was previously unknown.

Taken together, our pathway analyses suggest significant upregulation of tumor-promoting cascades by hypomethylation and disruption of tumor-suppressive functions by hypermethylation. While many of these genes/pathways have been previously implicated in cisplatin drug response/resistance [9], we also identified various cellular mediators (CIPT1A, alpha-integrins) previously unreported in the action of that widely used chemotherapeutic agent. Finally, although others have reported proteomic [27,28], chromosomal [29,30], gene expression [13,25,54], or histone/DNA modification [91] profiles of cancer cisplatin resistance, we believe this is the first report integrating aberrations in DNA methylation with changes in gene expression to identify likely drug sensitivity-associated biological pathways.

## Conclusion

This study establishes a valuable cell culture model system for the study of promoter CpG island DNA methylation aberrations related to the development of platinum resistance in ovarian cancer and their associated intracellular signaling pathways. We further demonstrate the value of rigorous bioinformatics analyses of integrating DNA methylation and gene expression profiles to elucidate epigenetically regulated pathways associated with the time-dependent acquisition of chemoresistance. Such experimental and computational approaches will be highly valuable for identifying key mediators of chemotherapy resistance as potential biomarkers or therapeutic targets.

## Abbreviations

DMH: differential methylation hybridization; CGI: CpG island; DNMT: DNA methyltransferase; DAVID: Database for Annotation, Visualization and Integrated Discovery; KEGG: Kyoto Encyclopedia of Genes and Genomes; GO: Gene Ontology; PI3K: phosphatidylinositol-3-kinase; CAM: cell adhesion molecule; 5-aza-dC: 5-aza-2'-deoxycytidine; GI_50_: 50% growth inhibition dose.

## Competing interests

The authors declare that they have no competing interests.

## Authors' contributions

ML prepared the manuscript and performed the gene expression assessments and all computational analyses (under the supervision of SK). JSM established the drug resistance culture model and performed the methylation microarray analyses, with supervision from PY and TH-MH. CB oversaw the project design, data interpretation, and final manuscript preparation. MJ performed the pathway validation and DNMT inhibitor assessments (under the supervision of JHC). KPN and SK oversaw the entire project and final manuscript preparation. TH-MH and KPN provided research funding for the study. All authors read and approved the final manuscript.

## Pre-publication history

The pre-publication history for this paper can be accessed here:



## Supplementary Material

Additional file 1 **Agilent annotation file for the customized 44 K promoter CpG island array**. The annotation file includes detailed information for microarray chip set-up and probe design for the customized 44 K promoter CpG island array used in the DMH experiment. Click here for file

Additional file 2 **Hierarchical clustering of the 3127 significantly up- or down-regulated probes in Round5**. The figure shows the clustering results with gene tree and probe names included of all significantly altered probes in Round5 vs. parental A2780 cells. Click here for file

Additional file 3 **Differentially expressed genes and probes in Round5 vs. parental A2780 cells**. The table lists all 2322 significantly differentially expressed genes, sorted by their average fold-changes, in Round5 vs. parental A2780 cells. Click here for file

Additional file 4 **Functional clustering analysis of all 1036 genes upregulated in Round5**. The table shows the functional annotation clustering analysis results of the upregulated genes in Round5 by DAVID. Functional annotation groups with geometric p-value less than 0.05 are listed. Each functional group contains related annotation terms that represent similar biological functions. Click here for file

Additional file 5 **Functional clustering analysis of all 1286 genes downregulated in Round5**. The table shows the functional annotation clustering analysis results of the downregulated genes in Round5 by DAVID. Functional annotation groups with geometric p-value less than 0.05 are listed. Each functional group contains related annotation terms that represent similar biological functions. Click here for file

## References

[B1] Ries LAG, Melbert D, Krapcho M, Stinchcomb DG, Howlader N, Horner MJ, Mariotto A, Miller BA, Feuer EJ, Altekruse SF (1975). SEER Cancer Statistics Review.

[B2] McGuire WP, Hoskins WJ, Brady MF, Kucera PR, Partridge EE, Look KY, Clarke-Pearson DL, Davidson M (1996). Cyclophosphamide and cisplatin compared with paclitaxel and cisplatin in patients with stage III and stage IV ovarian cancer. N Engl J Med.

[B3] Ozols RF (2005). Treatment goals in ovarian cancer. Int J Gynecol Cancer.

[B4] Agarwal R, Kaye SB (2003). Ovarian cancer: strategies for overcoming resistance to chemotherapy. Nat Rev Cancer.

[B5] Stewart DJ (2007). Mechanisms of resistance to cisplatin and carboplatin. Crit Rev Oncol Hematol.

[B6] Crul M, van Waardenburg RC, Beijnen JH, Schellens JH (2002). DNA-based drug interactions of cisplatin. Cancer Treat Rev.

[B7] Cepeda V, Fuertes MA, Castilla J, Alonso C, Quevedo C, Perez JM (2007). Biochemical mechanisms of cisplatin cytotoxicity. Anticancer Agents Med Chem.

[B8] Kelland L (2007). The resurgence of platinum-based cancer chemotherapy. Nat Rev Cancer.

[B9] Siddik ZH (2003). Cisplatin: mode of cytotoxic action and molecular basis of resistance. Oncogene.

[B10] Balch C, Huang TH, Brown R, Nephew KP (2004). The epigenetics of ovarian cancer drug resistance and resensitization. Am J Obstet Gynecol.

[B11] Feinberg AP, Tycko B (2004). The history of cancer epigenetics. Nat Rev Cancer.

[B12] Fojo T, Bates S (2003). Strategies for reversing drug resistance. Oncogene.

[B13] Jones PA, Baylin SB (2007). The epigenomics of cancer. Cell.

[B14] Konstantinopoulos PA, Fountzilas E, Pillay K, Zerbini L, Libermann TA, Cannistra SA, Spentzos D (2008). Carboplatin-induced gene expression changes in vitro are prognostic of survival in epithelial ovarian cancer. BMC Med Genomics.

[B15] Takai D, Jones PA (2002). Comprehensive analysis of CpG islands in human chromosomes 21 and 22. Proc Natl Acad Sci USA.

[B16] Balch C, Montgomery JS, Paik HI, Kim S, Huang TH, Nephew KP (2005). New anti-cancer strategies: epigenetic therapies and biomarkers. Front Biosci.

[B17] Brown R, Hirst GL, Gallagher WM, McIlwrath AJ, Margison GP, Zee AG van der, Anthoney DA (1997). hMLH1 expression and cellular responses of ovarian tumour cells to treatment with cytotoxic anticancer agents. Oncogene.

[B18] Cunningham JM, Christensen ER, Tester DJ, Kim CY, Roche PC, Burgart LJ, Thibodeau SN (1998). Hypermethylation of the hMLH1 promoter in colon cancer with microsatellite instability. Cancer Res.

[B19] Kitajima Y, Miyazaki K, Matsukura S, Tanaka M, Sekiguchi M (2003). Loss of expression of DNA repair enzymes MGMT, hMLH1, and hMSH2 during tumor progression in gastric cancer. Gastric Cancer.

[B20] Martini M, Ciccarone M, Garganese G, Maggiore C, Evangelista A, Rahimi S, Zannoni G, Vittori G, Larocca LM (2002). Possible involvement of hMLH1, p16(INK4a) and PTEN in the malignant transformation of endometriosis. Int J Cancer.

[B21] Dai W, Teodoridis JM, Graham J, Zeller C, Huang TH, Yan P, Vass JK, Brown R, Paul J (2008). Methylation Linear Discriminant Analysis (MLDA) for identifying differentially methylated CpG islands. BMC Bioinformatics.

[B22] Wei SH, Balch C, Paik HH, Kim YS, Baldwin RL, Liyanarachchi S, Li L, Wang Z, Wan JC, Davuluri RV (2006). Prognostic DNA methylation biomarkers in ovarian cancer. Clin Cancer Res.

[B23] Wei SH, Chen CM, Strathdee G, Harnsomburana J, Shyu CR, Rahmatpanah F, Shi H, Ng SW, Yan PS, Nephew KP (2002). Methylation microarray analysis of late-stage ovarian carcinomas distinguishes progression-free survival in patients and identifies candidate epigenetic markers. Clin Cancer Res.

[B24] Watts GS, Futscher BW, Holtan N, Degeest K, Domann FE, Rose SL (2008). DNA methylation changes in ovarian cancer are cumulative with disease progression and identify tumor stage. BMC Med Genomics.

[B25] Hsu DS, Balakumaran BS, Acharya CR, Vlahovic V, Walters KS, Garman K, Anders C, Riedel RF, Lancaster J, Harpole D (2007). Pharmacogenomic strategies provide a rational approach to the treatment of cisplatin-resistant patients with advanced cancer. J Clin Oncol.

[B26] Riedel RF, Porrello A, Pontzer E, Chenette EJ, Hsu DS, Balakumaran B, Potti A, Nevins J, Febbo PG (2008). A genomic approach to identify molecular pathways associated with chemotherapy resistance. Mol Cancer Ther.

[B27] Stewart JJ, White JT, Yan X, Collins S, Drescher CW, Urban ND, Hood L, Lin B (2006). Proteins associated with Cisplatin resistance in ovarian cancer cells identified by quantitative proteomic technology and integrated with mRNA expression levels. Mol Cell Proteomics.

[B28] Yan XD, Pan LY, Yuan Y, Lang JH, Mao N (2007). Identification of platinum-resistance associated proteins through proteomic analysis of human ovarian cancer cells and their platinum-resistant sublines. Journal of proteome research.

[B29] Prasad M, Bernardini M, Tsalenko A, Marrano P, Paderova J, Lee CH, Ben-Dor A, Barrett MT, Squire JA (2008). High definition cytogenetics and oligonucleotide aCGH analyses of cisplatin-resistant ovarian cancer cells. Genes, chromosomes & cancer.

[B30] Wasenius VM, Jekunen A, Monni O, Joensuu H, Aebi S, Howell SB, Knuutila S (1997). Comparative genomic hybridization analysis of chromosomal changes occurring during development of acquired resistance to cisplatin in human ovarian carcinoma cells. Genes, chromosomes & cancer.

[B31] Balch C, Yan P, Craft T, Young S, Skalnik DG, Huang TH, Nephew KP (2005). Antimitogenic and chemosensitizing effects of the methylation inhibitor zebularine in ovarian cancer. Molecular cancer therapeutics.

[B32] Fan M, Yan PS, Hartman-Frey C, Chen L, Paik H, Oyer SL, Salisbury JD, Cheng AS, Li L, Abbosh PH (2006). Diverse gene expression and DNA methylation profiles correlate with differential adaptation of breast cancer cells to the antiestrogens tamoxifen and fulvestrant. Cancer Res.

[B33] Yan PS, Wei SH, Huang TH (2002). Differential methylation hybridization using CpG island arrays. Methods Mol Biol.

[B34] Yan PS, Chen CM, Shi H, Rahmatpanah F, Wei SH, Huang TH (2002). Applications of CpG island microarrays for high-throughput analysis of DNA methylation. J Nutr.

[B35] Gentleman RC, Carey VJ, Bates DM, Bolstad B, Dettling M, Dudoit S, Ellis B, Gautier L, Ge Y, Gentry J (2004). Bioconductor: open software development for computational biology and bioinformatics. Genome Biol.

[B36] Benjamini Y, Hochberg Y (1995). Controlling the false discovery rate: A practical and powerful approach to multiple testing. Journal of the Royal Statistical Society Series B (Methodological).

[B37] Benjamini Y, Yekutieli D (2001). The control of the false discovery rate in multiple testing under dependency. Ann Stat.

[B38] Norris AW, Kahn CR (2006). Analysis of gene expression in pathophysiological states: balancing false discovery and false negative rates. Proc Natl Acad Sci USA.

[B39] Eisen MB, Spellman PT, Brown PO, Botstein D (1998). Cluster analysis and display of genome-wide expression patterns. Proc Natl Acad Sci USA.

[B40] Onto-Tools (The Intelligent Systems and Bioinformatics Laboratory). http://vortex.cs.wayne.edu/Projects.html.

[B41] Draghici S, Khatri P, Tarca AL, Amin K, Done A, Voichita C, Georgescu C, Romero R (2007). A systems biology approach for pathway level analysis. Genome Res.

[B42] KEGG: Kyoto Encyclopedia of Genes and Genomes. http://www.genome.jp/kegg/pathway.html.

[B43] Dennis G, Sherman BT, Hosack DA, Yang J, Gao W, Lane HC, Lempicki RA (2003). DAVID: Database for Annotation, Visualization, and Integrated Discovery. Genome Biol.

[B44] Huang da W, Sherman BT, Lempicki RA (2009). Systematic and integrative analysis of large gene lists using DAVID bioinformatics resources. Nat Protoc.

[B45] Database for Annotation, Visualization and Integrated Discovery (DAVID). http://david.abcc.ncifcrf.gov/.

[B46] Eva A, Robbins KC, Andersen PR, Srinivasan A, Tronick SR, Reddy EP, Ellmore NW, Galen AT, Lautenberger JA, Papas TS (1982). Cellular genes analogous to retroviral onc genes are transcribed in human tumour cells. Nature.

[B47] Behrens BC, Hamilton TC, Masuda H, Grotzinger KR, Whang-Peng J, Louie KG, Knutsen T, McKoy WM, Young RC, Ozols RF (1987). Characterization of a cis-diamminedichloroplatinum(II)-resistant human ovarian cancer cell line and its use in evaluation of platinum analogues. Cancer Res.

[B48] Gal-Yam EN, Saito Y, Egger G, Jones PA (2008). Cancer epigenetics: modifications, screening, and therapy. Annu Rev Med.

[B49] Cheng JC, Weisenberger DJ, Gonzales FA, Liang G, Xu GL, Hu YG, Marquez VE, Jones PA (2004). Continuous zebularine treatment effectively sustains demethylation in human bladder cancer cells. Mol Cell Biol.

[B50] Foubister V (2003). Drug reactivates genes to inhibit cancer. Drug Discov Today.

[B51] Ashburner M, Ball CA, Blake JA, Botstein D, Butler H, Cherry JM, Davis AP, Dolinski K, Dwight SS, Eppig JT (2000). Gene ontology: tool for the unification of biology. The Gene Ontology Consortium. Nat Genet.

[B52] Lebwohl D, Canetta R (1998). Clinical development of platinum complexes in cancer therapy: an historical perspective and an update. Eur J Cancer.

[B53] Lamendola DE, Duan Z, Yusuf RZ, Seiden MV (2003). Molecular description of evolving paclitaxel resistance in the SKOV-3 human ovarian carcinoma cell line. Cancer Res.

[B54] Sakamoto M, Kondo A, Kawasaki K, Goto T, Sakamoto H, Miyake K, Koyamatsu Y, Akiya T, Iwabuchi H, Muroya T (2001). Analysis of gene expression profiles associated with cisplatin resistance in human ovarian cancer cell lines and tissues using cDNA microarray. Hum Cell.

[B55] Li M, Paik HI, Balch C, Kim Y, Li L, Huang TH, Nephew KP, Kim S (2008). Enriched transcription factor binding sites in hypermethylated gene promoters in drug resistant cancer cells. Bioinformatics.

[B56] Rhee I, Bachman KE, Park BH, Jair KW, Yen RW, Schuebel KE, Cui H, Feinberg AP, Lengauer C, Kinzler KW (2002). DNMT1 and DNMT3b cooperate to silence genes in human cancer cells. Nature.

[B57] Robertson KD, Uzvolgyi E, Liang G, Talmadge C, Sumegi J, Gonzales FA, Jones PA (1999). The human DNA methyltransferases (DNMTs) 1, 3a and 3b: coordinate mRNA expression in normal tissues and overexpression in tumors. Nucleic Acids Res.

[B58] Mishra MV, Bisht KS, Sun L, Muldoon-Jacobs K, Awwad R, Kaushal A, Nguyen P, Huang L, Pennington JD, Markovina S (2008). DNMT1 as a molecular target in a multimodality-resistant phenotype in tumor cells. Mol Cancer Res.

[B59] Plumb JA, Strathdee G, Sludden J, Kaye SB, Brown R (2000). Reversal of drug resistance in human tumor xenografts by 2'-deoxy-5-azacytidine-induced demethylation of the hMLH1 gene promoter. Cancer Res.

[B60] Qiu YY, Mirkin BL, Dwivedi RS (2005). Inhibition of DNA methyltransferase reverses cisplatin induced drug resistance in murine neuroblastoma cells. Cancer Detect Prev.

[B61] Strathdee G, MacKean MJ, Illand M, Brown R (1999). A role for methylation of the hMLH1 promoter in loss of hMLH1 expression and drug resistance in ovarian cancer. Oncogene.

[B62] Ahluwalia A, Hurteau JA, Bigsby RM, Nephew KP (2001). DNA methylation in ovarian cancer. II. Expression of DNA methyltransferases in ovarian cancer cell lines and normal ovarian epithelial cells. Gynecol Oncol.

[B63] Ehrlich M, Woods CB, Yu MC, Dubeau L, Yang F, Campan M, Weisenberger DJ, Long T, Youn B, Fiala ES (2006). Quantitative analysis of associations between DNA hypermethylation, hypomethylation, and DNMT RNA levels in ovarian tumors. Oncogene.

[B64] Teodoridis JM, Hall J, Marsh S, Kannall HD, Smyth C, Curto J, Siddiqui N, Gabra H, McLeod HL, Strathdee G (2005). CpG island methylation of DNA damage response genes in advanced ovarian cancer. Cancer Res.

[B65] Xiong Y, Dowdy SC, Xue A, Shujuan J, Eberhardt NL, Podratz KC, Jiang SW (2005). Opposite alterations of DNA methyltransferase gene expression in endometrioid and serous endometrial cancers. Gynecol Oncol.

[B66] Wei SH, Brown R, Huang TH (2003). Aberrant DNA methylation in ovarian cancer: is there an epigenetic predisposition to drug response?. Annals of the New York Academy of Sciences.

[B67] Nyce J, Leonard S, Canupp D, Schulz S, Wong S (1993). Epigenetic mechanisms of drug resistance: drug-induced DNA hypermethylation and drug resistance. Proc Natl Acad Sci USA.

[B68] Nyce J, Liu L, Jones PA (1986). Variable effects of DNA-synthesis inhibitors upon DNA methylation in mammalian cells. Nucleic Acids Res.

[B69] Mortusewicz O, Schermelleh L, Walter J, Cardoso MC, Leonhardt H (2005). Recruitment of DNA methyltransferase I to DNA repair sites. Proc Natl Acad Sci USA.

[B70] Valinluck V, Sowers LC (2007). Endogenous cytosine damage products alter the site selectivity of human DNA maintenance methyltransferase DNMT1. Cancer Res.

[B71] ClinicalTrials.gov. http://clinicaltrials.gov/ct2/search.

[B72] Costello JF, Fruhwald MC, Smiraglia DJ, Rush LJ, Robertson GP, Gao X, Wright FA, Feramisco JD, Peltomaki P, Lang JC (2000). Aberrant CpG-island methylation has non-random and tumour-type-specific patterns. Nat Genet.

[B73] Matter K, Aijaz S, Tsapara A, Balda MS (2005). Mammalian tight junctions in the regulation of epithelial differentiation and proliferation. Curr Opin Cell Biol.

[B74] Matter K, Balda MS (2003). Signalling to and from tight junctions. Nat Rev Mol Cell Biol.

[B75] Morin PJ (2005). Claudin proteins in human cancer: promising new targets for diagnosis and therapy. Cancer Res.

[B76] Honda H, Pazin MJ, D'Souza T, Ji H, Morin PJ (2007). Regulation of the CLDN3 gene in ovarian cancer cells. Cancer Biol Ther.

[B77] Nakayama F, Semba S, Usami Y, Chiba H, Sawada N, Yokozaki H (2008). Hypermethylation-modulated downregulation of claudin-7 expression promotes the progression of colorectal carcinoma. Pathobiology.

[B78] Osanai M, Murata M, Chiba H, Kojima T, Sawada N (2007). Epigenetic silencing of claudin-6 promotes anchorage-independent growth of breast carcinoma cells. Cancer Sci.

[B79] Boireau S, Buchert M, Samuel MS, Pannequin J, Ryan JL, Choquet A, Chapuis H, Rebillard X, Avances C, Ernst M (2007). DNA-methylation-dependent alterations of claudin-4 expression in human bladder carcinoma. Carcinogenesis.

[B80] Litkouhi B, Kwong J, Lo CM, Smedley JG, McClane BA, Aponte M, Gao Z, Sarno JL, Hinners J, Welch WR (2007). Claudin-4 overexpression in epithelial ovarian cancer is associated with hypomethylation and is a potential target for modulation of tight junction barrier function using a C-terminal fragment of Clostridium perfringens enterotoxin. Neoplasia.

[B81] Cruet-Hennequart S, Maubant S, Luis J, Gauduchon P, Staedel C, Dedhar S (2003). alpha(v) integrins regulate cell proliferation through integrin-linked kinase (ILK) in ovarian cancer cells. Oncogene.

[B82] Maubant S, Cruet-Hennequart S, Poulain L, Carreiras F, Sichel F, Luis J, Staedel C, Gauduchon P (2002). Altered adhesion properties and alphav integrin expression in a cisplatin-resistant human ovarian carcinoma cell line. Int J Cancer.

[B83] Mazzarelli P, Pucci S, Bonanno E, Sesti F, Calvani M, Spagnoli LG (2007). Carnitine palmitoyltransferase I in human carcinomas: a novel role in histone deacetylation?. Cancer Biol Ther.

[B84] Pon YL, Auersperg N, Wong AS (2005). Gonadotropins regulate N-cadherin-mediated human ovarian surface epithelial cell survival at both post-translational and transcriptional levels through a cyclic AMP/protein kinase A pathway. J Biol Chem.

[B85] Hennessy BT, Smith DL, Ram PT, Lu Y, Mills GB (2005). Exploiting the PI3K/AKT pathway for cancer drug discovery. Nat Rev Drug Discov.

[B86] Zhang L, Huang J, Yang N, Greshock J, Liang S, Hasegawa K, Giannakakis A, Poulos N, O'Brien-Jenkins A, Katsaros D (2007). Integrative genomic analysis of phosphatidylinositol 3'-kinase family identifies PIK3R3 as a potential therapeutic target in epithelial ovarian cancer. Clin Cancer Res.

[B87] Dressman HK, Berchuck A, Chan G, Zhai J, Bild A, Sayer R, Cragun J, Clarke J, Whitaker RS, Li L (2007). An integrated genomic-based approach to individualized treatment of patients with advanced-stage ovarian cancer. J Clin Oncol.

[B88] Reimer D, Sadr S, Wiedemair A, Stadlmann S, Concin N, Hofstetter G, Muller-Holzner E, Marth C, Zeimet AG (2007). Clinical relevance of E2F family members in ovarian cancer – an evaluation in a training set of 77 patients. Clin Cancer Res.

[B89] Wang A, Li CJ, Reddy PV, Pardee AB (2005). Cancer chemotherapy by deoxynucleotide depletion and E2F-1 elevation. Cancer Res.

[B90] Chen G, Ghosh P, Osawa H, Sasaki CY, Rezanka L, Yang J, O'Farrell TJ, Longo DL (2007). Resistance to TGF-beta 1 correlates with aberrant expression of TGF-beta receptor II in human B-cell lymphoma cell lines. Blood.

[B91] Chekhun VF, Lukyanova NY, Kovalchuk O, Tryndyak VP, Pogribny IP (2007). Epigenetic profiling of multidrug-resistant human MCF-7 breast adenocarcinoma cells reveals novel hyper- and hypomethylated targets. Mol Cancer Ther.

